# Protective role of TLR9‐induced macrophage/microglia phagocytosis after experimental intracerebral hemorrhage in mice

**DOI:** 10.1111/cns.13919

**Published:** 2022-07-25

**Authors:** Jialiang Wei, Shuhui Dai, Chen Pu, Peng Luo, Yuefan Yang, Xiaofan Jiang, Xia Li, Wei Lin, Zhou Fei

**Affiliations:** ^1^ Department of Neurosurgery Xijing Hospital, Fourth Military Medical University Xi'an China; ^2^ Department of Health Service Fourth Military Medical University Xi'an China

**Keywords:** intracerebral hemorrhage, macrophage/microglia, phagocytosis, toll‐like receptor 9

## Abstract

**Introduction:**

Intracerebral hemorrhage (ICH) causes devastating morbidity and mortality, and studies have shown that the toxic components of hematomas play key roles in brain damage after ICH. Recent studies have found that TLR9 participates in regulating the phagocytosis of peripheral macrophages. The current study examined the role of TLR9 in macrophage/microglial (M/M) function after ICH.

**Methods:**

RAW264.7 (macrophage), BV2 (microglia), and HT22# (neurons) cell lines were transfected with lentivirus for TLR9 overexpression. Whole blood from C57BL/6 or EGFP^Tg/+^ mice was infused for phagocytosis and injury experiments, and brusatol was used for the experiments. Intraperitoneal injection of the TLR9 agonist ODN1826 or control ODN2138 was performed on days 1, 3, 5, 7, and 28 after ICH to study the effects of TLR9 in mice. In addition, clodronate was coinjected in M/M elimination experiments. The brains were collected for histological and protein experiments at different time points after ICH induction. Cellular and histological methods were used to measure hematoma/iron residual, M/Ms variation, neural injury, and brain tissue loss. Behavioral tests were performed premodeling and on days 1, 3, 7, and 28 post‐ICH.

**Results:**

Overexpression of TLR9 facilitated M/M phagocytosis and protected neurons from blood‐derived hazards in vitro. Furthermore, ODN1826 boosted M/M activation and phagocytic function, facilitated hematoma/iron resolution, reduced brain injury, and improved neurological function recovery in ICH mice, which were abolished by clodronate injection. The experimental results indicated that the Nrf2/CD204 pathway participated in TLR9‐induced M/M phagocytosis after ICH.

**Conclusion:**

Our study suggests a protective role for TLR9‐enhanced M/M phagocytosis via the Nrf2/CD204 pathway after ICH. Our findings may serve as potential targets for ICH treatment.

## Graphical abstract

Experimental Procedures: The WT C57BL/6 mouse or EGFP^Tg/+^ mouse whole blood was injected into BV2 and RAW264.7 cell lines with or without TLR9 overexpression for phagocytosis experiments. Male C57BL/6 mice were injected with autologous blood with or without clodronate liposome, and received TLR9 agonist ODN1826 or negative control ODN2138. The neurological, histological, and protein experiments were conducted at different time points after ICH modeling. Molecular Mechanism Diagram: TLR9 expression was upregulated after ICH in M/M and astrocyte. Post‐ICH, TLR9 activation facilitated M/M activation, enhanced M/M phagocytosis functions, and induced M/M polarization after ICH, which facilitated hematoma/iron clearance, alleviated neural injury, and improved functional recovery. Nrf2/CD204/HO‐1 pathways might play key role in TLR9‐induced M/M phagocytosis effect.

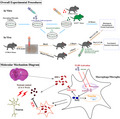

## 1 INTRODUCTION

Intracerebral hemorrhage (ICH) is the most severe subtype of stroke and has devastating consequences on families and society. However, there is no desirable treatment for ICH with an absolute benefit.^1^ Studies have shown that the toxic components of hematomas trigger immune cell activation as well as a variety of cytokines, chemokines, and free radical release, causing secondary brain injury, and reducing hematoma and/or its toxic components alleviates inflammation as well as injury after ICH.^2^, ^3^, ^4^, ^5^ Thus, many studies have focused on the pharmacological treatment of ICH,^6^ such as the enhancement of erythropagocytosis, mitigation of inflammation, and protection of white matter, but only a small portion of these studies showed promising results.^7^, ^8^, ^9^

Peripheral macrophages and resident microglia (macrophage/microglia, M/M) are important members of the innate immune system that play crucial roles in ICH. Studies have shown that M/Ms are activated at a very early stage post‐ICH and participate in hematoma clearance and neuroinflammation modulation.^10^ One of the major functions of M/Ms is to phagocytose cell debris and other clot components after ICH,^11^, ^12^ which helps neutralize the hematoma in a more integral way than targeting only one or some specific toxicants. Thus, interfering with M/Ms is a promising target for ICH treatment theoretically.^13^ However, the effective modulation of M/Ms, especially its phagocytic function, after ICH remains largely unknown.

The toll‐like receptors (TLRs) family of proteins plays a vital role in the innate immune system. It recognizes microbial infections or other danger signals and acts as a communicator with an adaptive immune system.^14^ One of its members, TLR9, is an important pathogen‐associated molecular pattern (PAMP) reactor that expresses primarily in B cells and plasmacytoid dendritic cells (pDCs). Furthermore, TLR9 recognizes bacterial and viral DNA with unmethylated cytosine–guanosine dinucleotide (CpG) motifs and participates in pathogen clearance.^15^ Recent studies have found that pharmacological agitation of TLR9 activates macrophages and enhances phagocytosis in certain physiopathological processes.^16^, ^17^ However, whether TLR9 is upregulated in the central nervous system after ICH and plays a role in hematoma clearance and neurorecovery remains unknown.

This study investigated TLR9 expression pattern and its role in M/Ms after ICH. The effects of TLR9‐induced M/M function on neural and functional recovery were examined.

## 2 MATERIALS AND METHODS

### 2.1 Animals

Animal experimental protocols were authorized by the Xijing Hospital Committee on the Use and Care of Animals. A total of 131 young male C57BL/6 mice (Jackson Laboratory) aged 2–3 months were sacrificed in our study. Three male C57BL/6 mice and three male enhanced green fluorescent protein (EGFP) transgene mice (EGFP^Tg/+^ mice) were used as blood donors for in vitro experiments. All mice were housed in a dichotomized light/dark cycle with controlled temperature and humidity and received water and food ad libitum. The odd/even number method was used for randomization. Briefly, mice were randomly marked with different numbers before certain interventions or treatments, and mice with even numbers were assigned to group A, while mice with odd numbers were assigned to group B. Mice exhibiting abnormal neurological scores pre‐ICH or dead after ICH were excluded from this study. All animal data reporting followed the ARRIVE 2.0 guidelines.^18^

### 2.2 ICH modeling

The mouse ICH model was constructed as described previously. Briefly, the mice were narcotized using ketamine (90 mg/kg, i.p.) and xylazine (5 mg/kg, i.p.), and body temperature were maintained at 37°C using a heating pad. Autologous blood was obtained from the right femoral artery before placing the mice in a stereotaxic frame (RWD; Shenzhen, China). A burr hole was drilled using dental instruments with the coordinates: 0.2 mm anterior, 2.5 mm lateral to the bregma, and 30 μl of blood were injected into the right caudate at a depth of 3.5 mm using 26‐gauge needles at a rate of 3 μl/min. The needles were kept in situ for another 10 minutes before retraction. Bone wax and anti‐infection sutures were used to seal the burr holes and skin, respectively.

### 2.3 Cell lines culture

The bone marrow‐derived macrophage cell line RAW264.7, microglial cell line BV2, and neuronal cell line HT22 were used in our study. All cell lines were cultured at 37°C in a 5% CO_2_ incubator with a culture medium composed of high‐glucose DMEM and 10% fetal bovine serum, and the culture medium was renewed every 2–3 days.

### 2.4 Lentivirus construction and transfection

The preparation of the TLR9 lentivirus has been described previously.^16^ Overexpression lentivirus was obtained from GeneChem Co. The multiplicity of the infection (MOI) is 100. PCR and western blotting were performed to further evaluate the transfection outcomes.

### 2.5 In vitro blood injury model

The C57BL/6 mouse whole blood was added to the culture medium of the cell lines before performing certain observations and interventions. For the M/M phagocytosis observation experiments, EGFP^Tg/+^ mice were used as blood donors. All cell lines were cultured in a 5% CO_2_ incubator at 37°C while undergoing blood‐induced injury.

### 2.6 Experimental groups

The in vitro experiment included three parts. In the first part, WT C57BL/6 mouse or EGFP^Tg/+^ mouse whole blood was injected into BV2‐OE, RAW264.7‐OE, BV2‐NC, and RAW264.7‐NC cell lines for different durations before carrying out the experiments. In the second part, the HT22 cell line received supernatant collected from the first part of the cell lines, and HT22‐OE and HT22‐NC were injected with mouse whole blood for injury investigation. In the third part, the BV2‐OE and RAW264.7‐IE cell lines were treated with certain concentrations of brusatol or equivalent DMSO for Nrf2 expression intervention.

The in vivo experiment included three parts. In the first part, male mice were euthanized on days 3 (*n* = 6) and 7 (*n* = 3) after ICH, and another three normal male mice were sacrificed as the pre‐ICH group. All brains were obtained for either protein or histological analysis (*n* = 3). In the second part, male ICH mice received the first 200 μg/kg ODN1826 injection intraperitoneally 2 hours after ICH modeling and received the same dose in the following 7 days consecutively. ODN2138 was used as a negative control. Mice were euthanized on days 1, 3, 5, 7, and 28 (*n* = 3 for the day 5 group and *n* = 11 for every other group). All the mice were sacrificed 6 h after receiving the final dose. The brains were collected for protein (*n* = 3) and histological analyses (*n* = 8). A total of 11 mice died within 24 h after ICH (five in the ODN2138 group and six in the ODN1826 group, *p* > 0.05). Third, male mice received a 30‐μl autologous blood injection combined with either 5 μl clodronate or control liposomes, and 200 μg/kg ODN1826 was injected as described previously. Two mice died within 24 h after ICH (one in the control liposome group and one in the clodronate group, *p* > 0.05). Mice were euthanized on day 7 (*n* = 6). All the brains were obtained for histological analysis.

### 2.7 Antibodies and drugs

The primary antibodies used in this study included polyclonal goat anti‐Iba1 (Abcam, 1:400), polyclonal rabbit anti‐Iba1 (Abcam, 1:400), polyclonal rabbit anti‐heme oxygenase‐1 (Enzo, 1:500), monoclonal mouse anti‐TLR9 (Abcam, 1:400), polyclonal rabbit anti‐TLR9 (GeneTex, 1:400), polyclonal goat anti‐GFAP (Abcam, 1:400), monoclonal rabbit anti‐Darpp‐32 (Cell Signaling, 1:100), polyclonal rabbit anti YM‐1 (Abcam, 1:200), monoclonal mouse anti‐NeuN (MERCK, 1:400), monoclonal mouse anti‐Arg1 (Santa Cruz, 1:200), polyclonal rabbit anti‐CD16 (Abcam, 1:200), polyclonal rabbit anti‐iNOS (Abcam, 1:200), polyclonal rabbit anti‐CD36 (GeenTex, 1:2000), monoclonal rabbit anti‐CD204 (Abcam, 1:2000), And polyclonal rabbit anti‐Nrf2 (Proteintech, 1:2000).

The secondary antibodies used in this article for immunofluorescence staining included donkey anti‐rabbit IgG (H + L) Alexa Fluor 488, donkey anti‐rabbit IgG (H + L) Alexa Fluor 594, donkey anti‐mouse IgG (H + L) Alexa Fluor 488, donkey anti‐mouse IgG (H + L) Alexa Fluor 594, donkey anti‐goat IgG (H + L) Alexa Fluor 488, and donkey anti‐goat IgG (H + L) Alexa Fluor 594, all obtained from Invitrogen.

The drugs used in this study included CpG ODN1826 and CpG ODN2138 (InvivoGen), clodronate liposomes, control liposomes (Liposoma Technology), and brusatol (Sigma‐Aldrich).

### 2.8 Perl's staining

Perl's staining kit (Solarbio) was used for iron staining, and staining was performed following the manufacturer's protocols.

### 2.9 Phagocytosis index calculation

The phagocytosis index was calculated using the following equation: GFP(+) cell count within Iba‐1(+) cell/DAPI count. GFP(+)/Iba‐1(+) cells indicate donor‐derived macrophages and were therefore excluded. The phagocytosis index indicated an average cell intake ability of RAW264.7 and BV2 cells.

### 2.10 Cell viability assay and lactate dehydrogenase (LDH) assay

The Cytotoxicity Detection Kit Plus (Roche Applied Bioscience) and Cell Counting Kit‐8 (CCK‐8; Dojindo) were used for LDH and cell viability assays, respectively, and the tests were performed as described previously.^19^

### 2.11 ELISA

The TNF‐α ELISA kit (Thermo Fisher Scientific) was used, and the assay was implemented following the manufacturer's protocols. The results were measured using a spectrophotometer at 450 nm as the primary wavelength.

### 2.12 Protein extraction and western blot analysis

Cultured cell lines and mouse brain tissue were lysed using RIPA buffer and protease inhibitor cocktail (Roche Applied Bioscience), and the brain tissue of ICH mice was collected separately as contralateral caudate, ipsilateral caudate (without hematoma clot), and hematoma clot. All protein samples were analyzed using a BCA assay kit (Biosynthesis Biotechnology).

Next, 20 μg protein from cell samples and 40 μg protein from tissue samples were applied when conducting western blot analysis. The detailed method was described previously.^19^

### 2.13 Brain histology staining

Mice were euthanized using pentobarbital (100 mg/kg), and their brains were collected via transcardiac perfusion with 4% paraformaldehyde (PFA) and were further fixed by immersion in PFA at 4°C overnight before dehydration using 30% glucose with 0.1 M phosphate‐buffered saline (PBS). The brains were sectioned coronally into 18 μm slices using a cryostat. Brain immunohistochemistry, immunofluorescence, and hematoxylin and eosin staining were performed as described previously.^20^ TUNEL staining was carried out following the manufacturer's protocol using the TUNEL kit (Beyotime, Shanghai, China).

### 2.14 Hematoma size measurement

The HE staining was used in the current study for hematoma size measurement. The slice with the largest clot size was measured as “hematoma area,” and the number of slices with hematoma was quantified as “*n*.” The hematoma size in each brain was calculated using the following equation: hematoma size (mm^3^) = hematoma area (mm^2^) × *n* × 0.018 (mm)/2.

### 2.15 Behavioral tests

Neurological deficits were assessed by recording the corner turn test and forelimb use asymmetry test results of all mice, as described previously.^21^ Briefly, the mice were placed in a corner at a 30° angle and the directions of turning were recorded 20 times. The results were obtained by calculating the percentage of right turns. For the forelimb asymmetry test, all the mice were observed for limb usage. The use of ipsilateral forelimb (I), contralateral forelimb (C), and both forelimbs (B) were recorded for the score calculation: forelimb use asymmetry score = (I−C)/(I + B + C). Spontaneous mouse movements were recorded in each group. Briefly, the times of vertical and horizontal movements within 2 min of each mouse were recorded, and all tasks were implemented by a blinded partner.

### 2.16 Cell counting

The cell counting was performed in a blinded manner. Three images at each area were pictured at 40× magnification and all counting was performed three times.

### 2.17 Statistical analysis

All observations and measurements were performed by blinded researchers. All values for each group are presented as mean ± SD. The Shapiro–Wilk test and/or Kolmogorov–Smirnov test were used for the data normality test. Statistical differences were analyzed according to different comparison situations by Student's *t*‐test, one‐way ANOVA, or two‐way ANOVA, as appropriate with Sidak's or Tukey's multiple comparisons test, and data that did not exhibit the normal/Gaussian distribution were analyzed using the Mann–Whitney test. Differences between certain groups were deemed statistically significant at *p <* 0.05. All materials and data needed for the conclusion assessment are displayed in the article and Supporting Information.

## 3 RESULTS

### 3.1 TLR9 expression elevated in macrophage and microglial cell lines and induced phagocytosis after blood injection

**FIGURE 1:**
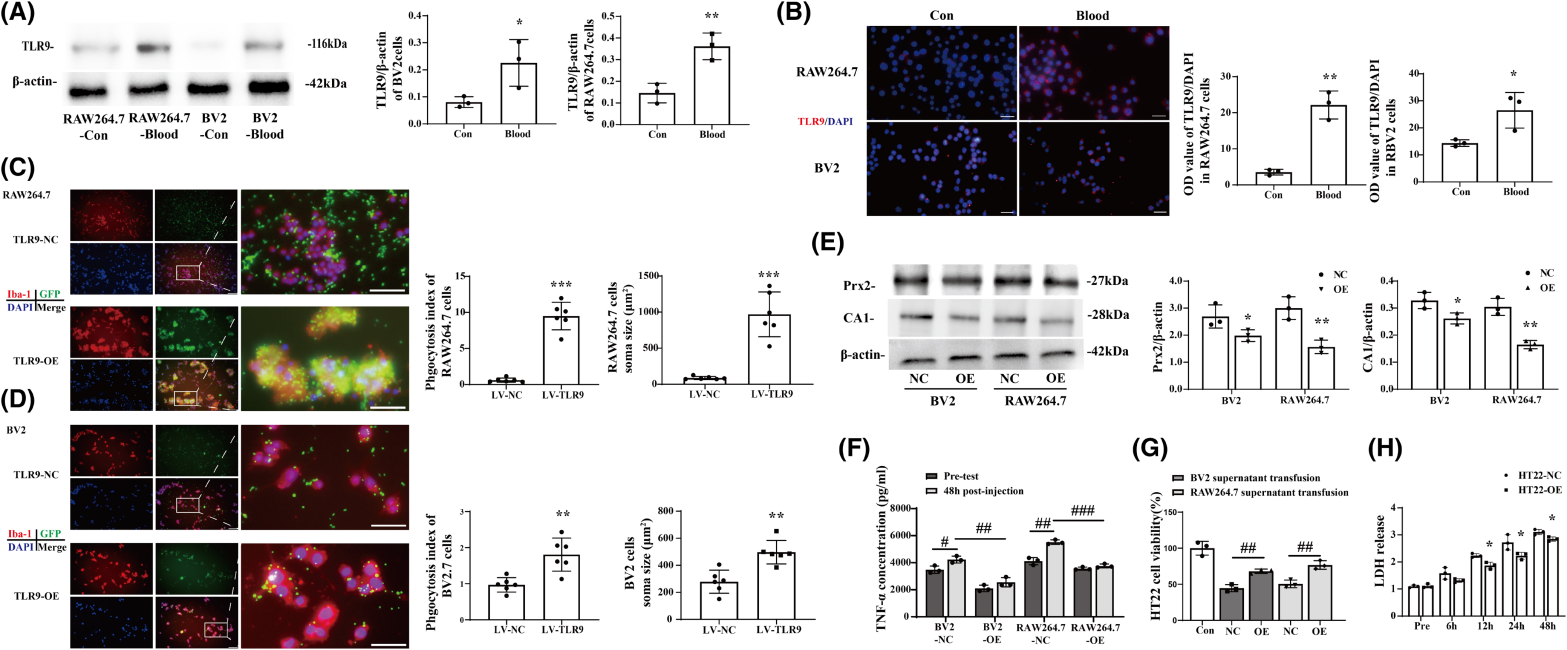
TLR9 expression elevated in macrophage and microglia cell lines and induced phagocytosis after blood infusion. (A) Western blot analysis of TLR9 protein level of BV2 and RAW264.7 cell lines 24 h after mouse whole blood injection, *n* = 3 for each group. (B) Immunofluorescence staining using TLR9 antibody in BV2 and RAW264.7 cell lines 24 h after mouse whole blood injection. Scale bar = 20 μm, *p < 0.05, **p < 0.01 vs. control group by Student's *t*‐test, *n* = 3 for each group. (C, D) Immunofluorescence staining using Iba‐1 (red) in RAW264.7 and BV2 cell lines 24 h after injecting whole blood obtained from EGFP^Tg/+^ mouse. The phagocytosis index and cell soma size were quantified in each group. Scale bars: low magnification = 20 μm, high magnification = 10 μm. ^#^ *p <* 0.05, ^##^ *p <* 0.01, ^###^ *p <* 0.001 vs. LV‐NC group by Student's *t*‐test. Values are mean ± SD, *n* = 6 for each group. (E) Western blot analysis of CA1 and Prx2 protein level of supernatant obtained from BV2 and RAW264.7 cell lines transfected with either NC or OE lentivirus 24 h after mouse whole blood injection. *p < 0.05, **p < 0.01 vs. NC group by Student's *t*‐test. (F) ELISA analysis of TNF‐α level of supernatant obtained from BV2 and RAW264.7 cell lines transfected with either NC or OE lentivirus before and 24 h after mouse whole blood injection. ^#^ *p <* 0.05, ^##^ *p <* 0.01, ^###^ *p <* 0.001 by two‐way ANOVA. (G) CCK‐8 assay of HT22 cell line transfused with different supernatant from blood‐cultured BV2 and RAW264.7 cell lines with or without TLR9 overexpression. (H) LDH release assay of HT22 with or without TLR9 overexpression 24 h after mouse whole blood injection. *p < 0.05, **p < 0.01 vs. NC group. ^#^ *p <* 0.05, ^##^ *p <* 0.01, ^###^ *p <* 0.001 by two‐way ANOVA. Values are mean ± SD, *n* = 3 for each group

Western blot analysis (TLR9/β‐actin: 0.081 ± 0.016 vs. 0.226 ± 0.070 in BV2 cell line, *p <* 0.05; 0.146 ± 0.037 vs. 0.361 ± 0.050 in RAW264.7 cell line, *p <* 0.01) (Figure 1A) and immunofluorescence (OD value/DAPI: 14.387 ± 1.011 vs. 26.504 ± 5.384 in BV2 cell line, *p <* 0.05; 3.533 ± 0.652 vs. 22.166 ± 3.162 in RAW264.7 cell line, *p <* 0.01) (Figure 1B) results showed that TLR9 expression was elevated in cultured RAW264.7 and BV2 cell lines after whole mouse blood injection. To test whether TLR9 participates in the phagocytosis of M/Ms, RAW264.7, and BV2, cells were transfected with lentivirus for TLR9 overexpression (OE) (Figure S1) before receiving a whole blood injection from EGFP^Tg/+^ mice. The results showed that after 24 h of injection, the OE cell lines engulfed more GFP (+) cells than the NC groups (Phagocytosis Index: 0.969 ± 0.183 vs. 1.808 ± 0.418 in BV2 cell lines, *p <* 0.01; 0.602 ± 0.263 vs. 9.499 ± 1.744 in RAW264.7 cell line, *p <* 0.001), and the soma size of OE cells was also larger than that of NC cells. This phenomenon was more obvious in RAW264.7 cells (278.427 ± 78.118 vs. 496.800 ± 79.305 μm^2^ in the BV2 cell line, *p <* 0.001; 86.726 ± 19.482 vs. 968.786 ± 283.070 μm^2^ in RAW264.7 cell line, *p <* 0.01). Notably, some RAW264.7 cells that overexpressed TLR9 exhibited drastic morphological changes in a multinucleated giant cell (MGC)‐like manner^20^ (Figure 1C,D).

### 3.2 TLR9 overexpression protected neurons from blood‐derived hazards

Western blot results showed that the protein level of toxicant blood component carbonic anhydrase 1 (CA1) (CA1/β‐actin: 0.328 ± 0.024 vs. 0.262 ± 0.017 in BV2 cell line, *p <* 0.05; 0.304 ± 0.026 vs. 0.165 ± 0.012 in RAW264.7 cell line, *p <* 0.01) and peroxiredoxin 2 (Prx2) (Prx2/β‐actin: 2.69 3 ± 0.352 vs. 1.981 ± 0.185 in BV2 cell line, *p <* 0.05; 3.050 ± 0.344 vs. 1.579 ± 0.208 in RAW264.7 cell line, *p <* 0.01) (Figure 1E) resided in the supernatant and the level of TNF‐α (4229.500 ± 206.443 vs. 2556.667 ± 276.860 pg/ml in BV2 cell line, *p <* 0.01; 5525.333 ± 136.563 vs. 3759.667 ± 116.076 pg/ml in RAW264.7 cell line, *p <* 0.001) (Figure 1F) secreted into supernatant were lower in TLR9‐OE RAW264.7 and BV2 cells group than that from NC group. In addition, results showed that HT22 cells that received supernatant from TLR9‐OE cell lines exhibited better cell viability than the NC group (44.594 ± 3.988% vs. 68.066 ± 2.428% in the BV2 supernatant transfusion group, *p <* 0.01; 50.468 ± 4.345% vs. 76.746 ± 4.815% in RAW264.7, *p <* 0.01) (Figure 1G). Blood caused less LDH release in TLR9 OE HT22 than NC groups at different time points (Read: 2.236 ± 0.060 vs. 1.867 ± 0.102 at 12 h, *p <* 0.05; 2.723 ± 0.228 vs. 2.230 ± 0.105 at 24 h, *p <* 0.05; 3.107 ± 0.824 vs. 2.848 ± 0.065 at 48 h, *p <* 0.05) (Figure 1H).

### 3.3 Brain TLR9 was upregulated after ICH in mice

**FIGURE 2:**
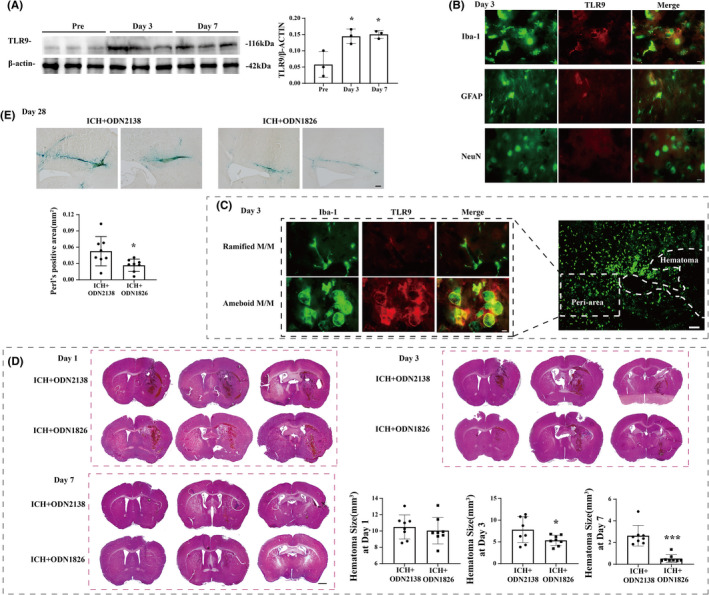
TLR9 participated in hematoma/iron clearance after ICH. (A) Western blot analysis of TLR9 protein level in ipsilateral basal ganglia of ICH mice at premodeling and day 3 and day7 after ICH. (B) Double labeling of TLR9 with GFAP (astrocyte marker), Iba‐1 (microglia marker), and NeuN (neuronal marker) in the ipsilateral basal ganglia at day 3 after ICH in WT mice. Scale bar = 10 μm. (C) Immunofluorescent double labeling of Iba‐1 (green) and TLR9 (red) in male mouse at day 3 after ICH. Scale bar: low magnification = 100 μm, high magnification = 10 μm. *p < 0.05 vs. premodeling group by one‐way ANOVA. Values are mean ± SD, *n* = 3 for each group. (D) HE staining of male mice brain after ICH at day 1, day 3, and day 7. The hematoma size of each group was calculated. Scale bar = 500 μm. *p < 0.05 vs. ICH + ODN2138 group by either two‐way ANOVA (at day 1 and day 3) or Mann–Whitney test (at day 7). (E) Perl's staining of male mice brain after ICH at day 28. The Perl's positive area was quantified in each group. Scale bar = 100 μm. *p < 0.05 vs. ICH + ODN2138 group by Student's *t*‐test. Values are mean ± SD, *n* = 8 for each group

Brain TLR9 expression in the ipsilateral basal ganglia (BG) was upregulated on days 3 and 7 after ICH, compared to pre‐ICH mouse brains (TLR9/β‐actin: 0.058 ± 0.034 pre‐ICH vs. 0.145 ± 0.029 in the 3‐day group, *p <* 0.05; 0.058 ± 0.034 pre‐ICH vs. 0.151 ± 0.011in the 7‐day group, *p <* 0.05) (Figure 2A). Double‐labeling immunofluorescence results showed that TLR9 immunoreactivity was colocalized with Iba‐1 (a microglial marker) and GFAP (an astrocyte marker), but barely colocalized with NeuN (a neuronal marker) (Figure 2B). In addition, immunofluorescence results showed that the amoeboid‐like Iba‐1(+) cells harbored stronger TLR9 immunoreactivity than ramified cells after ICH in the perihematomal area (Figure 2C).

### 3.4 TLR9 participated in hematoma/iron clearance, neural protection, and functional recovery after ICH

**FIGURE 3:**
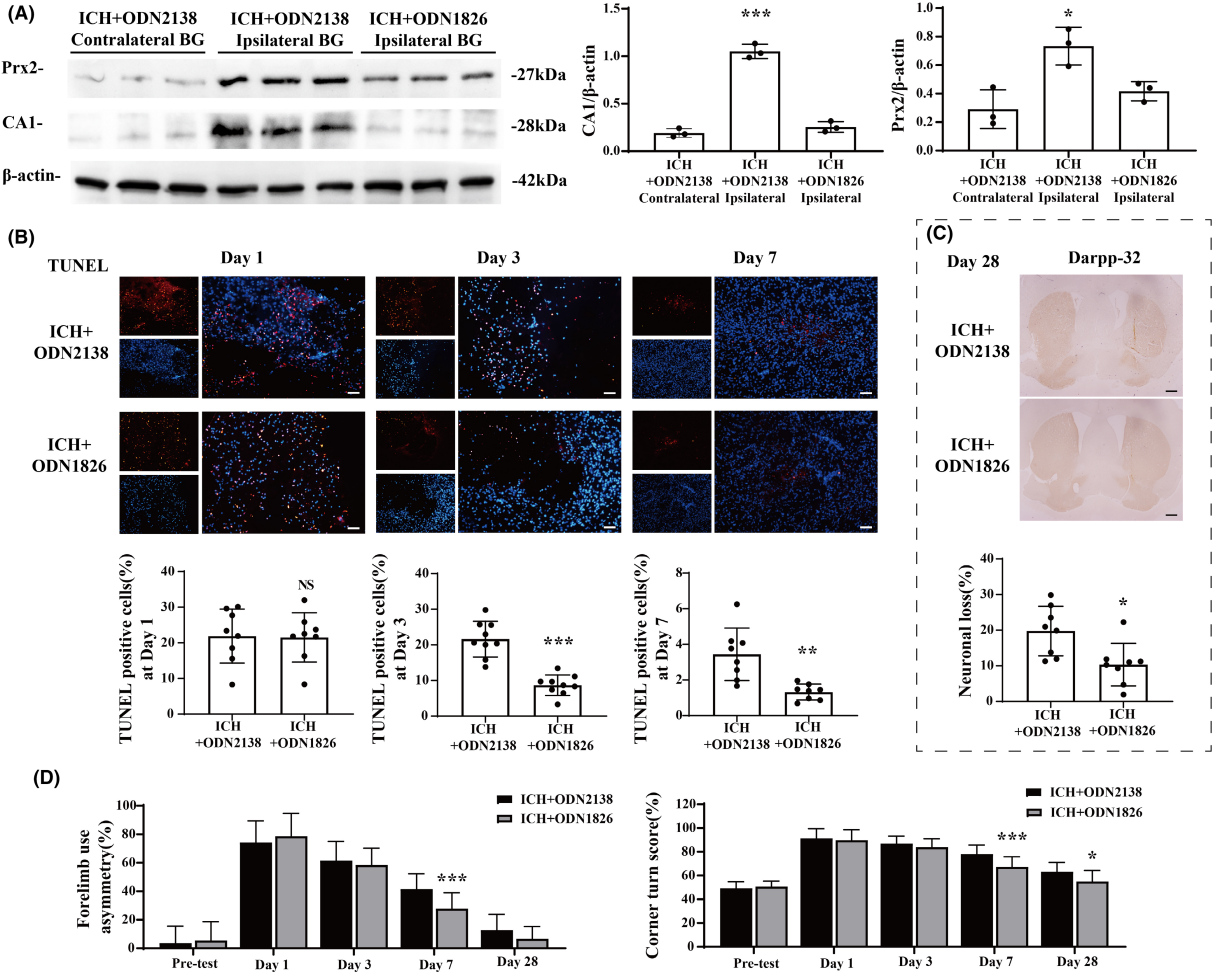
TLR9 activation alleviated neural injury and facilitated functional recovery after ICH. (A) Western blot analysis of CA1 and Prx2 protein levels in ipsilateral BG of ODN2138‐treated mice and ODN1826‐treated mice at day 3 after ICH. *n* = 3 for each group. *p < 0.05, ***p < 0.001 vs. other groups by one‐way ANOVA. (B) TUNEL staining (red) with DAPI (blue) of ODN2138‐treated mice and ODN1826‐treated mice ipsilateral BG at day 1, day 3, and day 7 after ICH, and the TUNEL‐positive cell percentages of each groups were quantified. *n* = 8 for each group. Scale bar = 100 μm. **p < 0.01, ***p < 0.001 vs. ICH + ODN2138 group by Student's *t*‐test. (C) Representative DARPP‐32 immunoreactivity of ODN2138‐treated mice and ODN1826‐treated mice at day 28 after ICH. *n* = 8 for each group. Scale bar = 500 μm. *p < 0.05 vs. ICH + ODN2138 group by Student's *t*‐test. (D) Forelimb use asymmetry and corner turn scores before and after ICH with ODN2138 or ODN1826 treatment. *n* = 44 for each group at day 1 and pretest, *n* = 33 for each group at day 3, *n* = 19 for each group at day 7, and *n* = 8 for each group at day 28. Values are mean ± SD. *p < 0.05, ***p < 0.001 vs. ICH + ODN2138 group by two‐way ANOVA

According to these results, our portfolio of ODN1826 use further activated TLR9 expression in ICH mice (Figure S2). The HE staining results showed that the mice that received ODN1826 injection had smaller hematoma sizes on day 3 (7.579 ± 2.925 vs. 5.343 ± 1.117 mm^3^, *p <* 0.05) and day 7 (2.631 ± 0.892 vs. 0.526 ± 0.337 mm^3^, *p <* 0.05), but not on day 1 after ICH ictus (10.496 ± 1.383 vs. 10.052 ± 1.529 mm^3^, *p* > 0.05) (Figure 2D). The iron residual at day 28 after ICH was also lower in mice that received ODN1826 injection than in IDN2138‐treated mice (0.053 ± 0.027 vs. 0.026 ± 0.011 mm^3^, *p <* 0.05) (Figure 2E); the protein levels of Prx2 (Prx2/β‐actin: 0.723 ± 0.121 vs. 0.447 ± 0.054, *p <* 0.05) and CA1 (CA1/β‐actin: 1.350 ± 0.061 vs. 0.226 ± 0.061, *p <* 0.001), which are harmful to the brain, were also lower in the ipsilateral BG of ODN1826‐treated mice than their ODN2138‐treated peers (Figure 3A). The TUNEL staining results showed that the neural death in ODN1826‐treated mice was less than that in the ODN2138‐treated group on day 3 and day 7 after ICH (21.646 ± 4.727% vs. 8.699 ± 2.714% on day 3, *p <* 0.001; 3.437 ± 1.474% vs. 1.317 ± 0.443% at day 7, *p <* 0.01) (Figure 3B), and the Darpp‐32(+) immunohistochemistry results indicated that the neuronal loss was less in ODN1826‐treated group at day 28 after ICH (19.768 ± 6.962% vs. 10.309 ± 5.970%, *p <* 0.05) **(**Figure 3C). The forelimb asymmetry test showed a better functional outcome in ODN1826‐treated mice on day 7 (41.600 ± 10.461 vs. 27.778 ± 10.830, *p <* 0.01) after ICH, and the corner turn test showed that ODN1826‐treated mice had better scores on day 7 (78.000 ± 7.616 vs. 67.222 ± 8.370, *p <* 0.001) and day 28 (63.182 ± 7.469 vs. 53.889 ± 8.749, *p <* 0.05) after ICH (Figure 3D). However, a significant spontaneous decrease was observed in ODN1826‐treated mice on day 1, day 3, and day 7 after ICH, which rose to normal on day 28 after ICH (Figure S3).

### 3.5 TLR9 activated M/Ms and boosted phagocytosis after ICH

**FIGURE 4:**
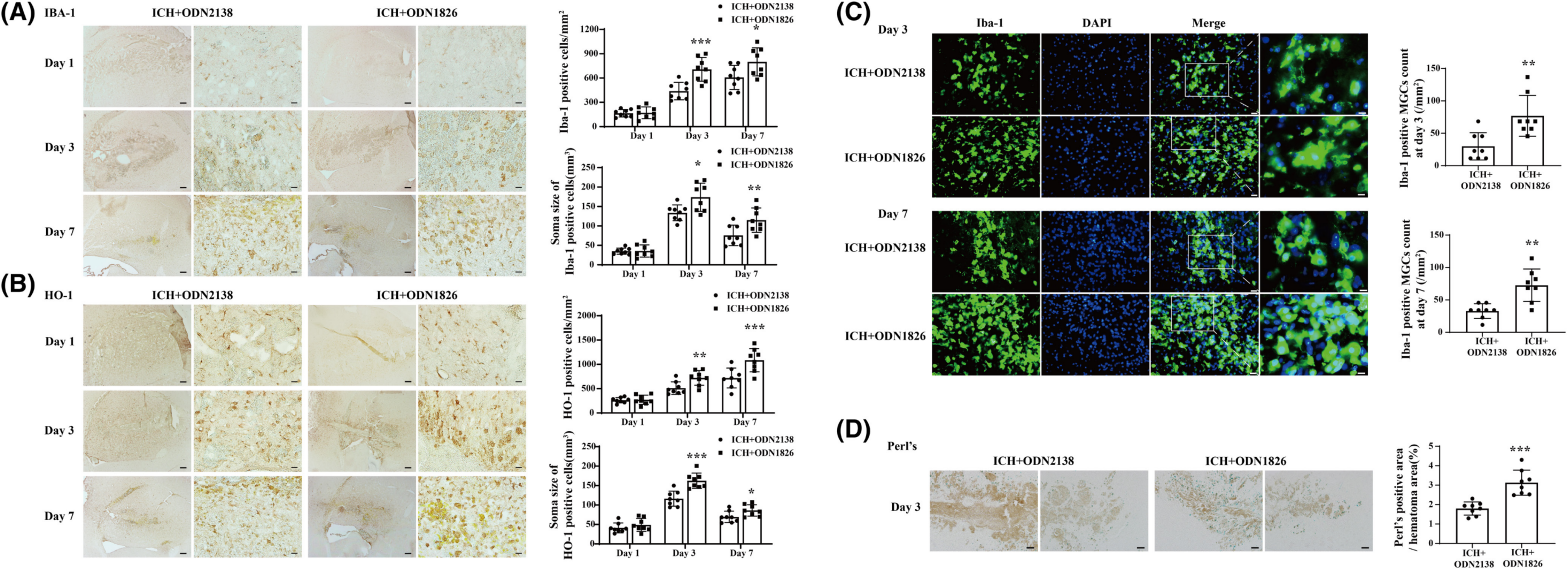
TLR9 induced M/Ms activation and MGCs formation. Positive cells of Iba‐1 (A) and HO‐1 (B) in perihematoma area at day 1, day 3, and day 7 after ICH treated with ODN2138 or ODN1826. The positive cell count and soma size were quantified in each group. Scale bar: low magnification = 200 μm, high magnification = 20 μm. *p < 0.05, **p < 0.01, ***p < 0.001 vs. ICH + ODN2138 group by two‐way ANOVA. *n* = 8 for each group. Values are mean ± SD. (C) Immunofluorescence staining using Iba‐1 and DAPI for MGCs quantification in male mice at day 3 and day 7 after ICH with ODN2138 or ODN1826 treatment. The high magnification images showed morphology of Iba‐1‐positive cells with multiple nuclei in the perihematomal area. Scale bar: low magnification = 20 μm, high magnification = 10 μm. (D) Perl's staining in male mice at day 3 after ICH with ODN2138 or ODN1826 treatment. Scale bar = 50 μm. **p < 0.01, ***p < 0.001 vs. ICH + ODN2138 group by Student's *t*‐test or by Mann–Whitney test (MGCs count at day 3). *n* = 8 for each group. Values are mean ± SD

We used several markers to identify M/M changes after ICH. Immunohistochemistry results showed that Iba‐1(+) and HO‐1(+) cell counts were higher in ODN1826‐treated mice than ODN2138‐treated mice at day 3 (Iba‐1: 421.800 ± 106.128 vs. 692.867 ± 135.290/mm^2^, *p <* 0.01; HO‐1: 534.533 ± 131.465 vs. 730.667 ± 137.654/mm^2^, *p <* 0.01) and day 7 (Iba‐1: 607.309 ± 143.961 vs. 811.300 ± 152.439/mm^2^, *p <* 0.05; HO‐1: 677.782 ± 179.354 vs. 1090.6 ± 148.151/mm^2^, *p <* 0.001) after ICH. The soma size of these positive cells were also larger in ODN1826‐treated mice than ODN2138‐treated ones (Iba‐1: 132.329 ± 18.301 vs. 171.808 ± 42.560 mm^2^ at day 3, *p <* 0.05; 71.399 ± 23.969 vs. 113.455 ± 29.854 mm^2^ at day 7, *p <* 0.01) (HO‐1: 116.601 ± 17.099 vs. 162.933 ± 17.316 mm^2^ at day 3, *p <* 0.001; 69.779 ± 13.942 vs. 85.595 ± 11.120 mm^2^ at day 7, *p <* 0.05) (Figure 4A,B). More Iba‐1(+) MGCs resided in the perihematoma area in the ODN1826‐treated group than in the ODN2138‐treated group on days 3 (29.925 ± 19.694 vs. 76.950 ± 29.481/mm^2^, *p <* 0.01) and 7 (32.776 ± 11.350 vs. 72.632 ± 25.091/mm^2^, *p <* 0.01) after ICH (Figure 4C). Perl's staining results showed a larger Perl's (+) area in the ODN1826‐treated mouse brain than in the ODN2138‐treated group (1.797 ± 0.315 vs. 3.129 ± 0.604%, *p <* 0.001) (Figure 4D).

### 3.6 TLR9 participated in M/Ms polarization after ICH

**FIGURE 5:**
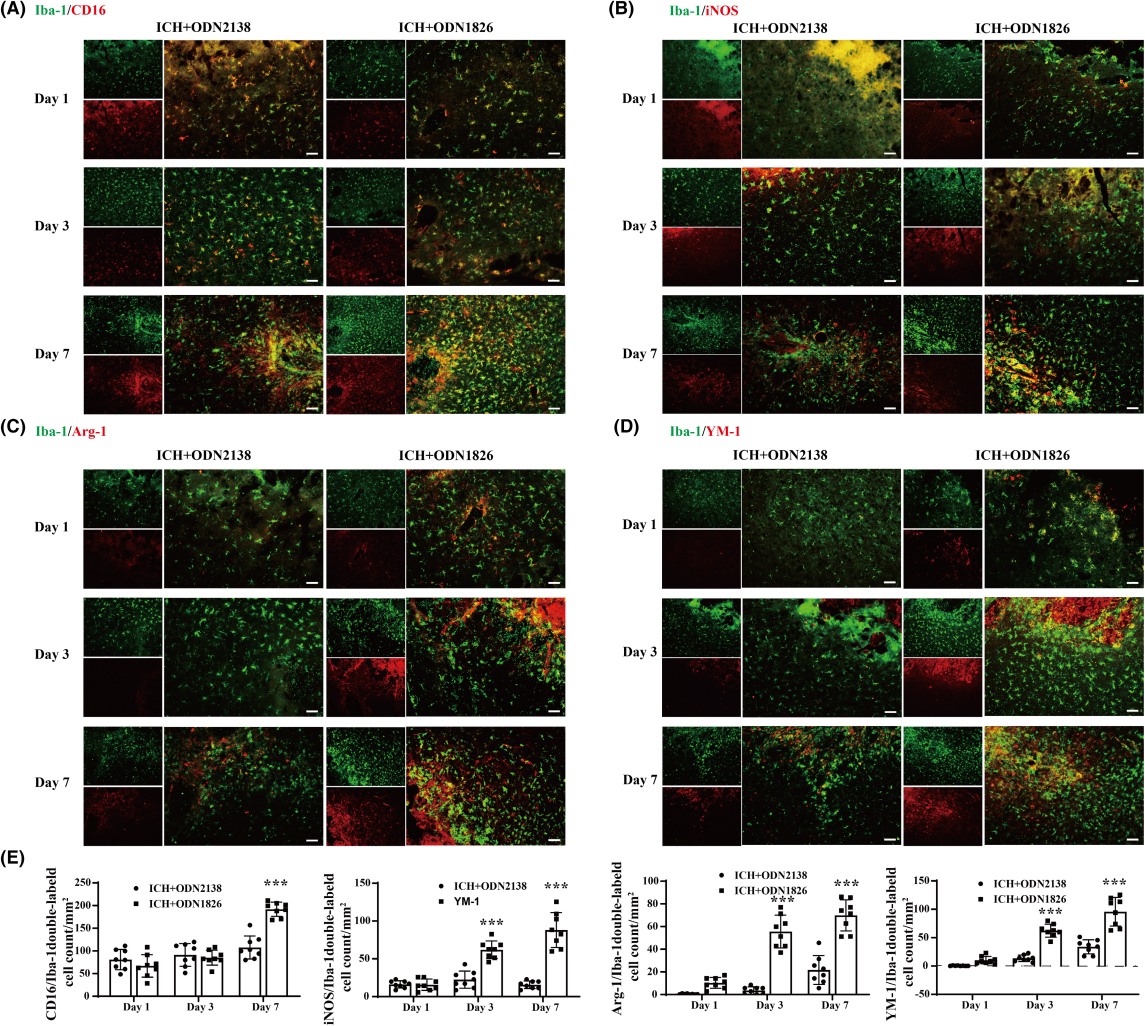
TLR9 participated in M/Ms polarization. (A) Immunofluorescence staining using Iba‐1 and CD16 in male mice at day 1, day 3, and day 7 after ICH with ODN2138 or ODN1826 treatment. (B) Immunofluorescence staining using Iba‐1 and iNOS in male mice at day 1, day 3, and day 7 after ICH with ODN2138 or ODN1826 treatment. (C) Immunofluorescence staining using Iba‐1 and Arg‐1 in male mice at day 1, day 3, and day 7 after ICH with ODN2138 or ODN1826 treatment. (D) Immunofluorescence staining using Iba‐1 and YM‐1 in male mice at day 1, day 3, and day 7 after ICH with ODN2138 or ODN1826 treatment. (E) Quantification of double‐labeled cell count in each groups at all three time points. Scale bar = 50 μm. *n* = 8 for each group. Values are mean ± SD. **p < 0.01, ***p < 0.001 vs. ICH + ODN2138 group by two‐way ANOVA

The immunofluorescence results showed that the CD16(+)/Iba‐1(+) M1 cell count increased in ODN1826‐treated mice on day 7 (108.707 ± 20.034 vs. 191.790 ± 14.717/ mm^2^, *p <* 0.001) after ICH (Figure 5A), and the iNOS(+)/Iba‐1(+) M1 cell count increased in ODN1826‐treated mice on day 3 (22.444 ± 10.614 vs. 61.988 ± 10.922/mm^2^, *p <* 0.001) and day 7 (15.675 ± 4.726 vs. 88.098 ± 21.597/mm^2^, *p <* 0.001) after ICH (Figure 5B). Meanwhile, the Arg‐1(+)/Iba‐1(+) M2 cell count was increased in ODN1826‐treated mice at days 3 and 7 (3.483 ± 2.937 vs. 54.783 ± 12.941/mm^2^ at day 3, *p <* 0.001; 23.837 ± 11.090 vs. 70.300 ± 10.873/mm^2^ at day 7, *p <* 0.001) after ICH (Figure 5C), and the YM‐1(+)/Iba‐1(+) M2 cell count showed similar results (13.538 ± 5.284 vs. 62.344 ± 10.705/mm^2^ at day 3, *p <* 0.001; 33.844 ± 11.615 vs. 95.688 ± 23.695/mm^2^ at day 7, *p <* 0.001) (Figure 5D).

### 3.7 Clodronate liposome reversed the ODN1826‐induced hematoma clearance, brain injury alleviation, M/Ms activation, and functional recovery

**FIGURE 6:**
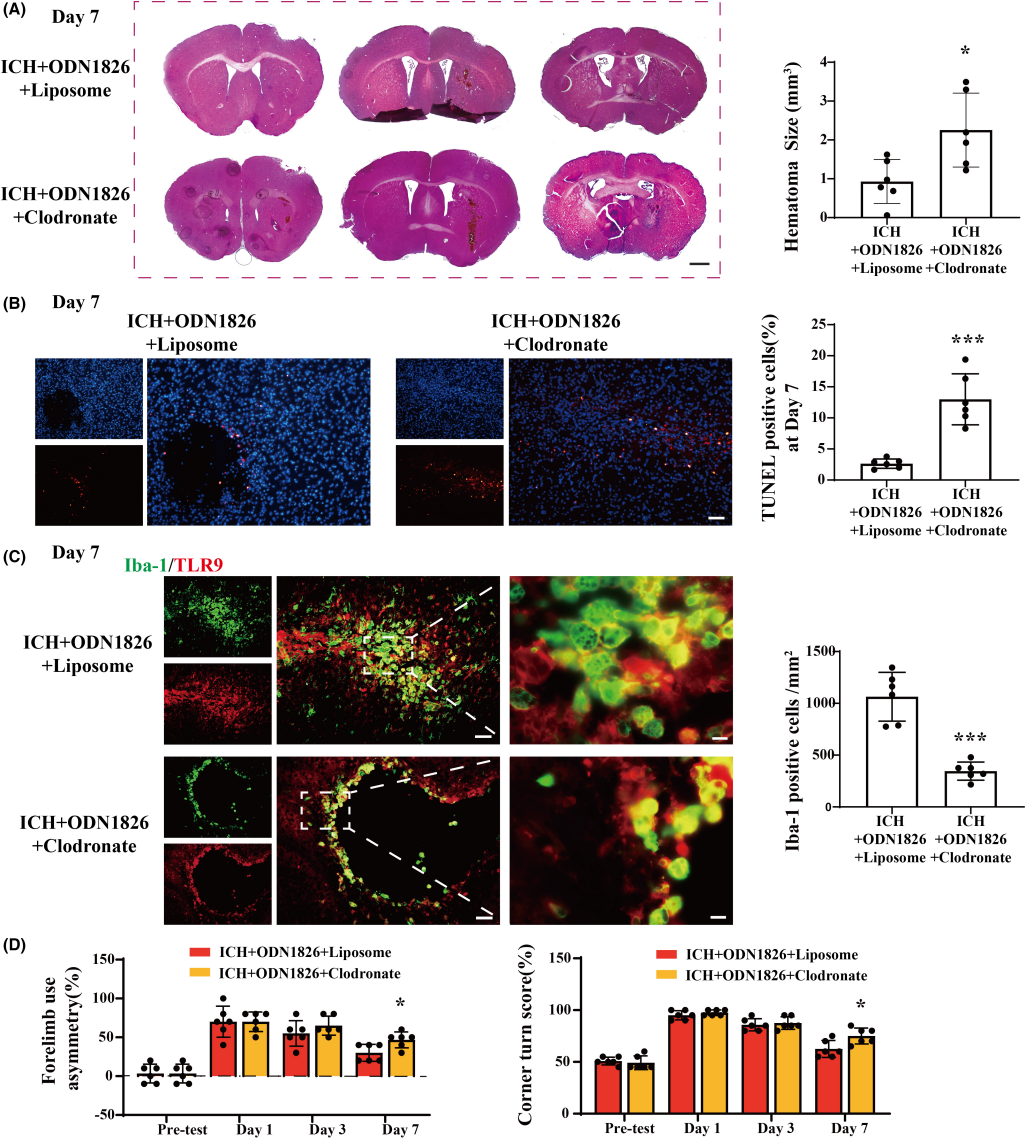
Clodronate liposome abolished the TLR9‐induced neuroprotection effect after ICH. (A) HE staining of male mice brain after ICH with ODN1826 + clodronate or ODN1826 + liposome interventions at day 7. The hematoma size of each group was calculated. Scale bar = 500 μm. **p < 0.01 vs. ICH + ODN1826 + liposome group by Student's *t*‐test. (B) TUNEL staining (red) with DAPI (blue) of ODN1826 + clodronate‐treated mice and ODN1826 + control liposome‐treated mice ipsilateral BG at day 7 after ICH, and the TUNEL‐positive cell percentage of each group were quantified. Scale bar = 50 μm. ***p < 0.001 vs. ICH + ODN1826 + liposome group by Student's *t*‐test. (C) Immunofluorescence staining using Iba‐1 and TLR9 at day 7 after ICH with ODN1826 + clodronate or ODN1826 + control liposome treatment. Scale bar: low magnification = 50 μm, high magnification = 10 μm. ***p < 0.001 vs. ICH + ODN1826 + liposome group by Student's *t*‐test. (D) Forelimb use asymmetry and corner turn scores before and after ICH with ODN1826 + clodronate or ODN1826 + liposome treatment. *p < 0.05, **p < 0.01, ***p < 0.001 vs. ICH + ODN1826 + liposome group. *n* = 6 for each group. Values are mean ± SD

Clodronate liposomes were coinjected with blood to eliminate M/Ms. The hematoma size on day 7 after ICH was higher in clodronate‐treated mice than in control liposome‐treated mice (2.420 ± 0.742 vs. 0.929 ± 0.517 mm^3^ on day 7, *p <* 0.05) (Figure 6A). In addition, the TUNEL‐positive cell count re‐increased in clodronate‐treated mice compared to that in control liposome‐treated mice at day 7 post‐ICH (12.989 ± 3.752% vs. 2.641 ± 0.689%, *p <* 0.001) (Figure 6B). The immunofluorescence staining results showed that Iba‐1(+) M/Ms were significantly lower in the brains of clodronate‐treated mice than in the control group (345.800 ± 79.981 vs. 1064.000 ± 215.061/mm^2^ at day 7, *p <* 0.001), and the vast majority of the remaining Iba‐1(+) M/Ms also expressed TLR9 and exhibited amoeboid‐like morphology (Figure 6C). The corner turn test and forelimb asymmetry test were performed, and the results showed that ICH mice receiving clodronate injection had worse functional outcomes than the control group on day 7 after ICH (corner turn: 75.000 ± 7.071 vs. 62.500 ± 7.500, *p <* 0.001; forelimb asymmetry: 46.667 ± 9.428 vs. 30.000 ± 10.000, *p <* 0.001) (Figure 6D).

### 3.8 TLR9 facilitated M/Ms functional changes via Nrf2/CD204/HO‐1 signaling pathway

**FIGURE 7:**
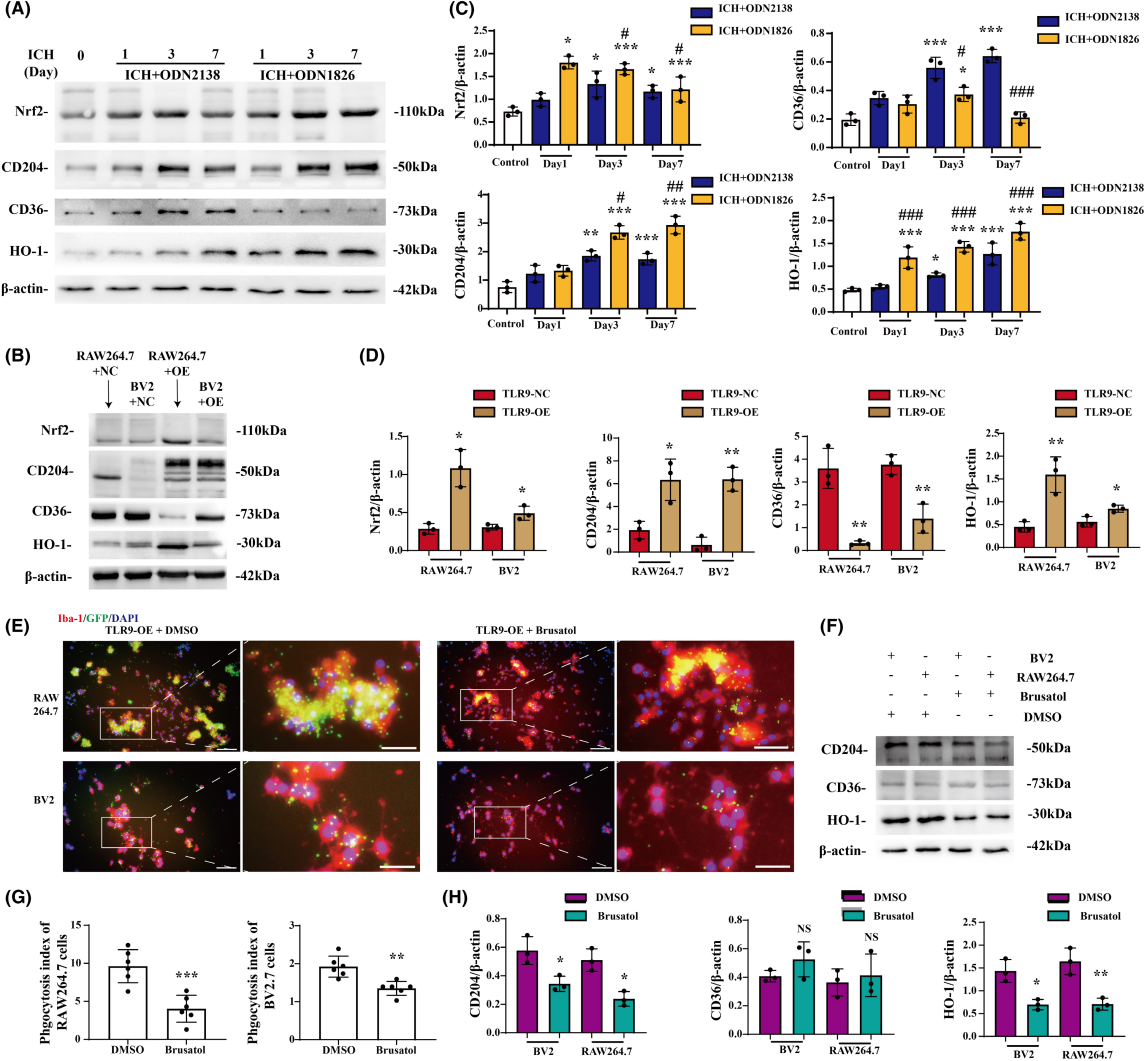
Nrf2/CD204/HO‐1 pathway participated in TLR9‐induced M/M phagocytosis. (A) Representative western blots of Nrf2, CD204, CD36, and HO‐1 expression in the ipsilateral basal ganglia tissue of ICH mice with ODN2138 or ODN1826 treatment. (B) Representative western blots of Nrf2, CD204, CD36, and HO‐1 expression in the blood‐injected BV2 and RAW264.7 cell lines with or without TLR9 overexpression. (C) The relative protein expression level quantification of mice in different groups. *p < 0.05, **p < 0.01, ***p < 0.001 vs. control group, and ^#^ *p <* 0.05, ^##^ *p <* 0.01, ^###^ *p <* 0.001 vs. ICH + ODN2138 group by two‐way ANOVA. Values are mean ± SD, *n* = 3 for each group. (D) The relative quantification of protein expression in different cell line groups. *p < 0.05, **p < 0.01 vs. TLR9‐NC group by two‐way ANOVA. Values are mean ± SD, *n* = 3 for each group. (E) Immunofluorescence staining using Iba‐1 (red) in TLR9‐overexpressed, DMSO, or brusatol pretreated RAW264.7 and BV2 cell lines 24 hours after injecting whole blood obtained from EGFP^Tg/+^ mouse. (F) Representative western blots of CD204, CD36, and HO‐1 expression in TLR9‐overexpressed, DMSO, or brusatol pretreated RAW264.7 and BV2 cell lines 24 h after whole blood injection. (G) The phagocytosis indices were quantified in each group. Scale bars: low magnification = 20 μm, high magnification = 10 μm. **p < 0.001, ***p < 0.001 vs. DMSO‐treated group by Student's *t*‐test. (H) The relative quantification of protein expression in different groups. *p < 0.05, **p < 0.01 vs. DMSO‐treated group by two‐way ANOVA. Values are mean ± SD, *n* = 3 for each group

The signaling pathways involved in the prophagocytic effects of TLR9 after ICH were further investigated. According to the western blot results, Nrf2 and HO1 expression was upregulated after ICH and was further enhanced by TLR9 activation. Furthermore, CD204, also known as the type I macrophage scavenger receptor (MSR1), was remarkably upregulated after TLR9 activation, whereas the expression of CD36, a type II macrophage scavenger receptor (MSR2), exhibited the opposite tendency (Figure 7A,C). Similar results were obtained in the in vitro study (Figure 7B,D). We then used Nrf2‐specific inhibitor brusatol in TLR9‐overexpressed BV2 and RAW264.7 cell lines to further investigate the potential mechanism of TLR9‐mediated M/Ms changes after ICH (Figure S4). The western blot results showed that the pharmacological inhibition of Nrf2 in both cell lines downregulated CD204 and HO‐1 expression levels, but not CD36 expression (Figure 7F,H). Also, the Nrf2 inhibition significantly blocked the TLR9‐induced phagocytosis boosting effects in both BV2 and RAW264.7 cell lines (phagocytosis index: 1.923 ± 0.252 vs. 1.350 ± 0.168 in BV2 cell line, *p <* 0.001; 9.633 ± 1.988 vs. 4.031 ± 1.625 in RAW264.7 cell line, *p <* 0.01) (Figure 7E,G).

## 4 DISCUSSION

This study found that (1) TLR9 expression in macrophage and microglial cell lines enhanced phagocytosis and mediated neural protection after blood injection; (2) TLR9 expression was upregulated after ICH in M/M and astrocytes; (3) post‐ICH TLR9 activation facilitated hematoma/iron clearance, alleviated neural injury, and improved functional recovery; (4) post‐ICH TLR9 activation facilitated M/M activation, enhanced M/M phagocytosis functions, and induced M/M polarization after ICH, which were reversed by M/M depletion via clodronate liposome injection; and (5) the Nrf2/CD204/HO‐1 pathway participated in TLR9‐induced M/M phagocytosis.

Unlike other TLRs families, such as TLR4 and TLR2,^22^, ^23^ the role of TLR9 after ICH remains unknown. Studies have shown that TLR9 is expressed in microglia, astrocytes, and neurons under various pathophysiological conditions in the central nervous system.^24^, ^25^, ^26^ In line with these results, in our study, TLR9 expression was increased in cultured microglia and macrophages after mouse blood injection. The total expression of TLR9 also increased in the mouse caudate after ICH and was expressed mainly in the microglia and astrocytes 3 days after ICH ictus. However, in this study, neurons barely expressed TLR9 after ICH. This discrepancy may be due to different pathological models, and hence, the unparallel mechanism beneath. This study found that TLR9 immunoreactivity colocalized more with large amoeboid‐like M/Ms than with small, ramified M/Ms. Since our previous study showed that amoeboid‐like M/Ms phagocytosed more red blood cells (RBCs) than ramified ones,^20^ this result indicated a potential relationship between TLR9 and M/Ms phagocytosis.

Furthermore, M/Ms are major intrinsic scavengers that play important roles in engulfing cell debris and toxic components. Previous studies have shown the significance of these two cells in ICH.^27^, ^28^ This study showed that TLR9 could effectively facilitate phagocytosis in M/M cell lines and reduce potential toxicants derived from blood that might harm neurons, indicating the potential neuroprotective effects of TLR9 activation. The in vitro results indicate that TLR9 might be a feasible target for regulating M/M phagocytosis after ICH. Thus, we further explored its in vivo function by injecting the TLR9 activator ODN1826 into mice post‐ICH. Histological experiments showed that ODN1826‐induced TLR9 activation substantially promoted Iba‐1‐ and HO‐1‐positive cell numbers with an enlarged cell body size around the hematoma, which indicated the M/M activation effects of TLR9.

Studies and our previous research have shown that interventions targeting M/M could effectively activate M/Ms and enhance MGCs formation, which is involved in hematoma clearance and brain injury alleviation after ICH.^20^, ^21^ Similarly, in this study, TLR9‐overexpressed RAW264.7, cells exhibited MGC‐like morphological changes that were similar to MGCs seen in human and mouse brains after ICH,^20^, ^29^ and more MGCs were seen in ODN1826‐treated mice after ICH. In addition, prx2 and CA1, two major toxicants released from the hematoma, were remarkably lower on day 3 in ODN1826‐treated mice brains, and the ICH mouse brain on day 3 showed more hemosiderin in the ODN1826‐treated group than in the control group. These results indicate the phagocytosis‐boosting function of TLR9. Recent studies have shown that perihematomal iron concentration assessed by T2*‐weighted MRI is a sensitive biomarker for ICH injury.^30^, ^31^ In this study, Perl's staining was used for iron quantification and the results indicated less iron deposition in the ODN1826‐treated group than that in the control group. Further studies using the T2* method are needed to precisely calculate iron concentration. TLR9‐mediated protection was further evidenced by the fact that ODN1826‐treated mice exhibited faster hematoma resolution, less neural injury, and better functional outcomes than control mice after ICH. The clodronate liposome‐mediated M/Ms decrease counteracted ODN1826‐induced hematoma resolution, neural protection, and functional recovery effects. These results suggest a beneficial effect of TLR9‐induced M/M phagocytosis on ICH.

Polarization of M/M occurs after ICH and varies according to the microenvironment.^32^ It has been proven that M1 polarization is proinflammatory, which facilitates the inflammatory cascade and thus hazards the ICH brain, and M2 polarization is anti‐inflammatory, which helps in neural recovery.^33^ Although this dichotomized categorization was somehow conceived of absoluteness that simplified the orchestrated roles of M/Ms, it is pragmatic to comprehend the framework of M/M functioning.^10^ In this study, we used some classic markers to elucidate the M/M polarization pattern after TLR9 activation. Our results showed that TLR9 facilitated M1 and M2 polarization within 7 days after ICH. Moreover, some M1 and M2 cells exhibited amoeboid‐like morphology and were located in the perihematomal zone, which is in line with our previous study.^15^ These results indicate the complex effects of TLR9‐mediated M/M polarization after ICH.

As a transcriptional factor, Nrf2 was found to play a key role in various engulfing processes, such as autophagy, micropinocytosis, and macrophage phagocytosis,^34^, ^35^, ^36^ and was upregulated in hemorrhagic diseases, such as subarachnoid hemorrhage.^37^ Our current study showed that Nrf2 was upregulated in ICH and further enhanced after TLR9 activation, indicating a potential relationship between these two molecules under ICH conditions. The macrophage scavenger receptor (MSR) is expressed primarily on myeloid cells and participates in immune homeostasis maintenance and macrophage uptake.^38^, ^39^ Recently, MSR was found to participate in macrophage phagocytosis after stroke.^40^, ^41^ In this study, CD204 (MSR1) and CD36 (MSR2) were upregulated after ICH, CD204 expression was further enhanced, and CD36 was downregulated by TLR9 activation. Furthermore, Nrf2/CD204 activation was at least partially responsible for TLR9‐induced M/M phagocytosis, since inhibition of Nrf2 downregulated CD204 expression and reversed TLR9‐mediated M/M functional changes. Our results indicate a novel TLR9‐related pathway after ICH.

This present study dosed mice 2 h after ICH ictus, which is theoretically plausible and clinically feasible. According to our results, mice that received the ODN1826 injection exhibited better neurological function recovery, as demonstrated by functional tests. However, mice that received ODN1826 injection presented transient but remarkable spontaneous activity decrease within 7 days after ICH, which implied that our portfolio of ODN1826 usage needs further adjustment or that the systematic activation of TLR9 might cause transient but unneglectable negative effects.

In conclusion, our results demonstrate that post‐ICH TLR9 activation can enhance M/M activation and phagocytic function, which facilitates hematoma/iron clearance and reduces brain injury in mice. This study identified a potential novel target for ICH treatment.

There are limitations to this study: (1) Only one strategy is used in this study, further studies are required to determine the optimal dosage. (2) The effect of ODN1826 has only been explored in young male mice, and future studies should investigate its effect in aged and female animals. (3) We only observed brain damage and recovery within 28 days post‐ICH, and whether TLR9 activation could influence long‐term brain changes requires further investigation. (4) The neuroinflammation changes, especially cytokine secretion variation solicited by TLR9 activation after ICH, require further investigation.

## AUTHOR CONTRIBUTIONS

JLW performed most of the animal experiment and drafted the manuscript. SHD performed the in vitro experiment. CP collected and analyzed the data. PL participated in animal behavioral tests. YFY participated in the data analysis. XFJ and XL revised the manuscript. WL and ZF conceived the study idea and supervised the study.

## CONFLICT OF INTEREST

All authors declare that they have no conflict of interest.

## Funding information

This study was supported by the National Natural Science Foundation of China (No. 82101374, No. 81771239, No. 81974188, and No. 81901186).

## Supporting information


Figures S1–S4

## Data Availability

All materials and data needed for the conclusion assessment are displayed in the manuscript and Supporting Information.
