# Supplementary material for: Protective role of TLR9‐induced macrophage/microglia phagocytosis after experimental intracerebral hemorrhage in mice
Source: CNS Neurosci Ther. 2022 Jul 25;28(11):1800–13. doi: 10.1111/cns.13919 (PMC9532915; doi:10.1111/cns.13919)
Supplement: Supplementary file 1 — Figures S1–S4 [file CNS-28-1800-s001.pdf]

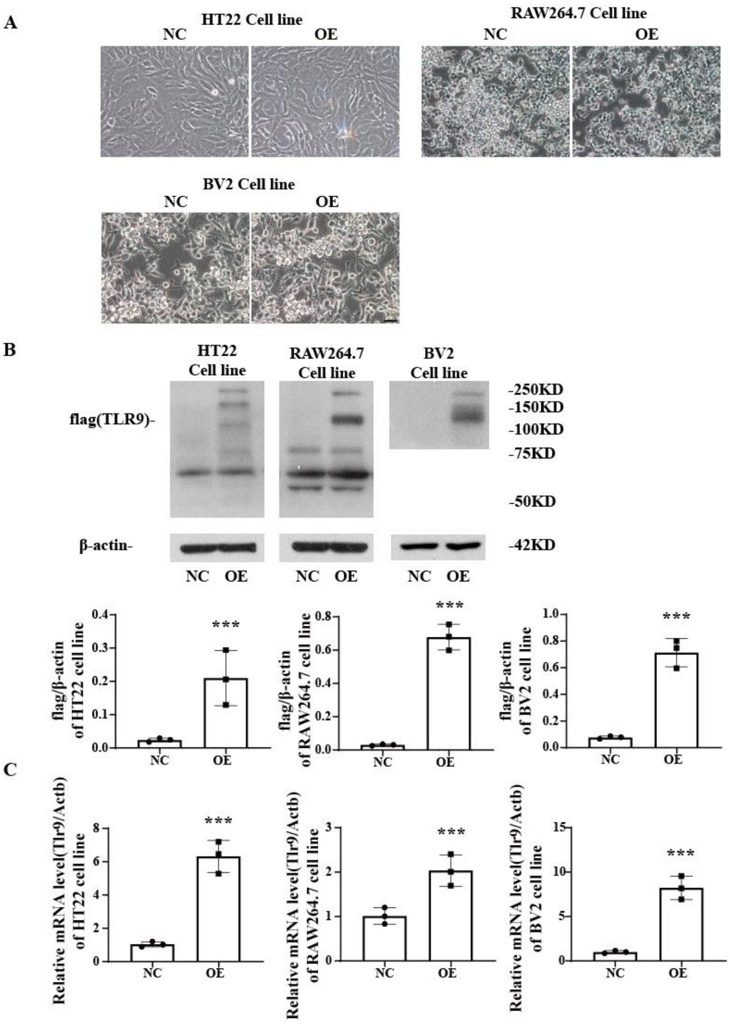

Figure S1. TLR9 overexpression cell lines verification. **A.** Morphology observation of BV2, RAW264.7 and HT22 cell lines with NC or OE lentivirus transfection. Scale bar=50μm. **B.** Western blot for Flag expression level of all three cell lines with different lentivirus. **c.** qPCR for relative TLR9 mRNA level of all three cell lines with different lentivirus. n=8 for each group. Values are mean ± SD. ****P*<0.001 vs LV-NC group by student t test.

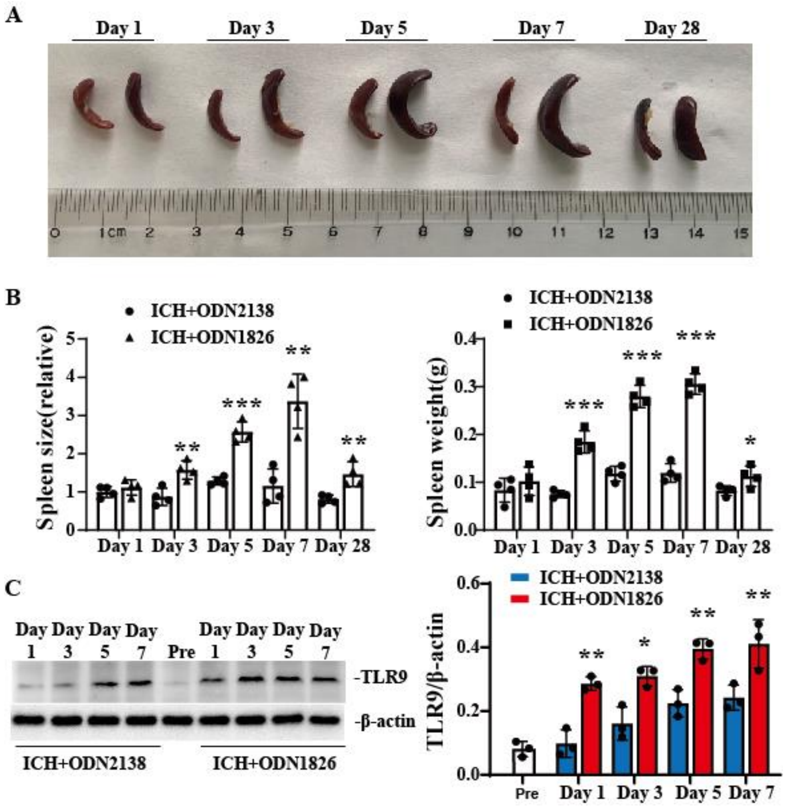

Figure S2. TLR9 activation of pharmacological intervention. **A.** Measurement of spleen size of male mice at day 1, day 3, day 5, day 7 and day 28 after ICH with ODN2138 or ODN1826 treatment. **B.** Spleen size and weight quantification of male mice at day 1, day 3, day 5, day 7 and day 28 after ICH with ODN2138 or ODN1826 treatment. n=8 for each group. **C.** Western blot for TLR9 expression in ipsilateral BG of mice received ODN2138 or ODN1826 treatment at day 1, day 3, day 5 and day 7 after ICH. n=3 for each group. Values are mean ± SD. **P*<0.05, ***P*<0.01, ****P*<0.001 vs ICH+ODN2138 group by two-way ANOVA.

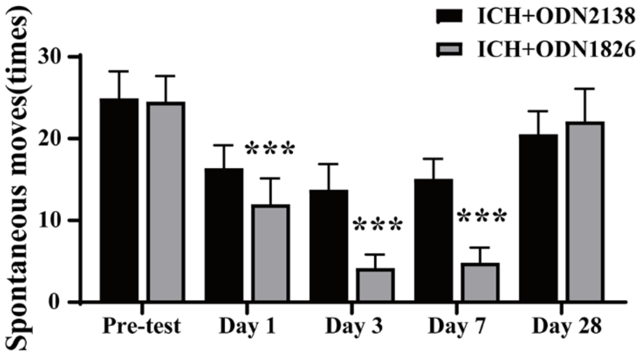

Figure S3. Spontaneous movement of ICH mice of each group. n=44 for each group at day 1 and pre-test, n=33 for each group at day 3, n=19 for each group at day 7 and n=8 for each group at day 28. Values are mean ± SD. ****P*<0.001 vs ICH+ODN2138 group by two-way ANOVA.

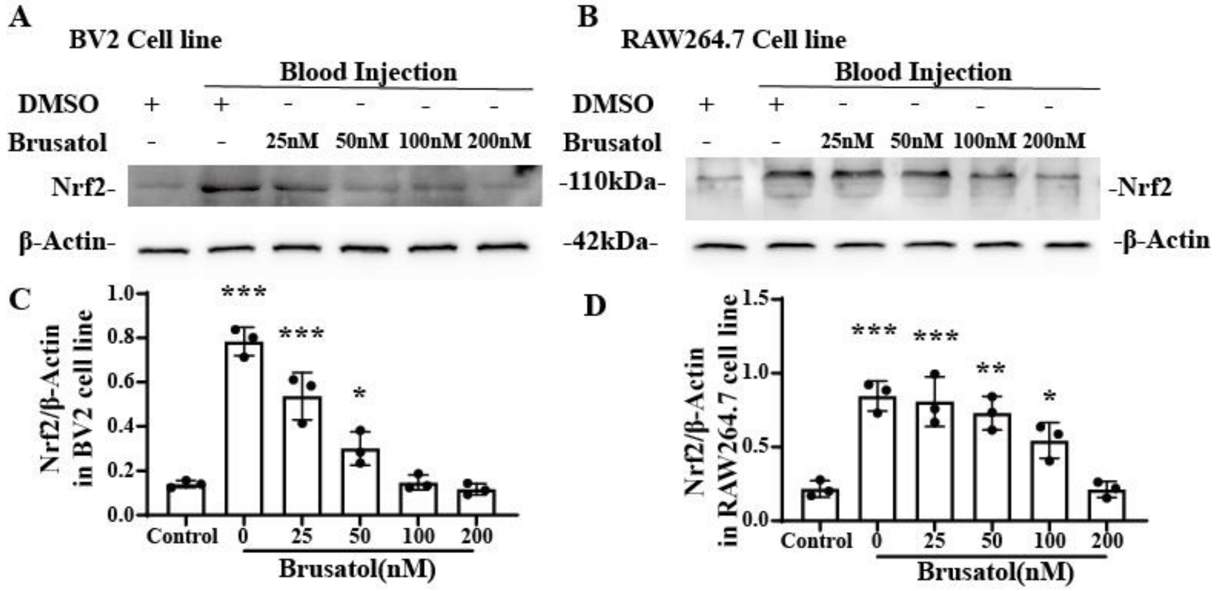

Figure S4. Brusatol concentration for Nrf2 inhibition in BV2 and RAW264.7 cell lines. **A.** Western blot for Nrf2 expression level under different Brusatol concentration in BV2 cells. **B.** Western blot for Nrf2 expression level under different Brusatol concentration in RAW264.7 cells. n=3 for each group. Values are mean ± SD. **P*<0.05, ***P*<0.01, ****P*<0.001 vs control group by two-way ANOVA.
